# Evidence for positive selection of taurine genes within a QTL region on chromosome X associated with testicular size in Australian Brahman cattle

**DOI:** 10.1186/1471-2156-15-6

**Published:** 2014-01-10

**Authors:** Russell E Lyons, Nguyen To Loan, Leanne Dierens, Marina R S Fortes, Matthew Kelly, Sean S McWilliam, Yutao Li, Rowan J Bunch, Blair E Harrison, William Barendse, Sigrid A Lehnert, Stephen S Moore

**Affiliations:** 1 CSIRO, Animal, Food and Health Sciences, Queensland Bioscience Precinct, Brisbane, Qld 4067, Australia; 2 School of Chemistry and Molecular Biosciences, The University of Queensland, Brisbane, Qld 4067, Australia; 3 Queensland Alliance for Agriculture and Food Innovation, Centre for Animal Science, The University of Queensland, Brisbane, Qld 4072, Australia

**Keywords:** Bovine, Reproductive trait, Candidate gene, Haplotype analysis, Association study

## Abstract

**Background:**

Previous genome-wide association studies have identified significant regions of the X chromosome associated with reproductive traits in two *Bos indicus*-influenced breeds: Brahman cattle and Tropical Composites. Two QTL regions on this chromosome were identified in both breeds as strongly associated with scrotal circumference measurements, a reproductive trait previously shown to be useful for selection of young bulls. Scrotal circumference is genetically correlated with early age at puberty in both male and female offspring. These QTL were located at positions 69–77 and 81–92 Mb respectively, large areas each to which a significant number of potential candidate genes were mapped.

**Results:**

To further characterise these regions, a bioinformatic approach was undertaken to identify novel non-synonymous SNP within the QTL regions of interest in Brahman cattle. After SNP discovery, we used conventional molecular assay technologies to perform studies of two candidate genes in both breeds. Non-synonymous SNP mapped to Testis-expressed gene 11 (*Tex11*) were associated (*P* < 0.001) with scrotal circumference in both breeds, and associations with percentage of normal sperm cells were also observed (*P* < 0.05). Evidence for recent selection was found as *Tex11* SNP form a haplotype segment of *Bos taurus* origin that is retained within Brahman and Tropical Composite cattle with greatest reproductive potential.

**Conclusions:**

Association of non-synonymous SNP presented here are a first step to functional genetic studies. Bovine species may serve as a model for studying the role of *Tex11* in male fertility, warranting further in-depth molecular characterisation.

## Background

Indicine cattle *(Bos indicus)* are socially- and economically-important breeds globally and dominate beef production systems in tropical and sub-tropical regions, and are an important resource in developing nations for food security in vulnerable communities [1]. Presence of indicine cattle is largely due to these breeds being better able to tolerate high temperature and humid environments common in these regions. *Bos indicus* also have improved tolerance to ticks and other tropical challenges compared to taurine breeds (*Bos taurus*) commonly found in temperate regions [2,3]. However, it is well documented that indicine breeds are inferior in terms of reproductive efficiency in comparison to taurine cattle [4-6]. For example, Brahman bulls typically reach puberty at a later age compared to taurine breeds, with downstream impacts on a number of economically-important indicators of herd productivity and profitability including generation interval and calving rates [7-9]. Tropically-adapted composite herds have also been developed with the aim of retaining the beneficial adaptations of indicine breeds for environmental stressors, while integrating taurine genes for production traits including reproductive performance [10-12]. Improving reproductive performance of indicine cattle will provide positive impacts in terms of both food security and environmental impacts through increased efficiency in livestock production in areas where hunger is an issue and demand for animal protein is increasing.

Fertility of individual sires is an important production and economic trait affecting reproductive performance of the whole herd. However, fertility is not an easily defined trait, comprising a variety of important heritable traits for selection of early maturing animals [13]. Whereas estimates of heritability for female fertility traits are low, heritability estimates for testicular traits are moderate to high and therefore can be used for effective selection [13-15]. For example, scrotal circumference (SC) is an easy to measure and consistent indicator trait for puberty in bulls with heritability across breeds ranging from medium to high [16]. Also, SC is favourably correlated to other traits affecting both male and female reproductive performance, including sperm quality, percentage of normal sperm, semen concentration, body weight, age at first pregnancy, progesterone levels and age at puberty in heifers [15-24]. Genetic selection that uses SC measurements to improve male fertility can impact positively on whole herd reproductive performance. Likewise, percentage of normal sperm (PNS) impacts directly on bull fertility, as it is a meaningful predictor of calf output per bull [25]. Therefore, measurements of SC and PNS are used as indicators in breeding programs for selecting fertile bulls [18,20].

A limitation in the use of SC and PNS as selection tools is that these reproductive traits cannot be measured before bulls reach 12–24 months of age. Time constraint can limit the attractiveness of these traits as selection tools. Identifying genetic markers associated with these traits would potentially overcome this limitation as they can be measured in DNA samples extracted from blood at birth.

Recent genome-wide association studies (GWAS) in Brahman and Tropical Composite cattle has resulted in the identification of a number of genomic regions associated with SC, PNS and other reproductive traits [26-30]. Fortes et al. [29] reported a 30 Mb region from approximately 62 to 92 Mb positions on chromosome X associated with SC in Brahmans. This region was also associated with the age at which a bull achieves a scrotal circumference of 26 cm (AGE26), regarded as puberty [27]. This finding was confirmed in a subsequent study on a Tropical Composite population [30], with significant polymorphisms grouped in two discrete regions at 69–77 Mb and 81–92 Mb positions on the X chromosome.

These confirmed QTL regions warrant further investigation and in the current study, two candidate genes, androgen receptor (*AR*) and testis expressed 11 (*Tex11*), were investigated for their association with SC and PNS measurements in Brahman and Tropical Composite populations. Androgen Receptor was chosen for its proximity to the QTL peak and its well-studied roles during embryogenesis and puberty for male phenotype development and sexual maturation [31]. Androgens, principally 5α-dihydrotestosterone (DHT) and testosterone, are essential for the maintenance of behaviour and function of male reproduction and exert their effects by interacting with AR. Both native ligands of this receptor, testosterone and DHT bind to AR to activate target gene expression at the transcriptional level. Androgen-AR activity results in the promotion of maintenance and development of male reproduction and male phenotype [31]. The *Tex11* gene is also in the same QTL region in chromosome X [29,30], and has previously been reported to be essential for male meiosis and fertility in humans and pigs [32,33].

The aim of this study was to characterise the variations in these genes including 5′UTR, test their association with male reproductive traits and investigate the origin of favourable haplotypes. Precedence for the importance of polymorphisms in the 5′UTR gene region include SNP within this region of the bovine growth hormone receptor gene, which has a marked effect on beef production traits [34], and the association of a SNP within the 5′UTR of the bovine lactoferrin (*LF*) gene with reproductive parameters and uterine infection in dairy cattle [35]. Identification of novel single nucleotide polymorphisms (SNP) markers was possible through a combination of bioinformatic analyses of genome sequence data of representative Brahmans and target sequence analysis within the study population. Discovered markers were used to test these candidate genes for their association with observed phenotypic variation of SC and PNS across both Brahman and Tropical Composite populations. Haplotypes across the X chromosome were determined to be of *Bos taurus* or *Bos indicus* origin. Haplotypes from across the entire X chromosome were contrasted with *Tex11* haplotypes to verify the origin of haplotypes and search for evidence of recent selection.

## Results

### SNP discovery using Brahman genomes

Genome sequences from 16 Brahman bulls, sires of the studied population, were initially compared to the published *Bos taurus* UMD3.1 Bovine Genome Assembly on chromosome X between positions 60,168,532 and 93,999,806 bp. This region has previously been shown to contain 2 QTL associated with SC, PNS and other reproductive traits in both Brahman and Tropical Composite bulls [26-30]. Using Samtools [36], 139,409 SNP were identified, including 247 non-synonymous SNP (nsSNP) affecting 95 genes of which 192 SNP (78%) have not been previously reported in the dbSNP short genetic variations database (NCBI). All new SNP have been entered into dbSNP.

Bioinformatic analysis initially identified 555 SNP across the ~210 kb region of the *AR* genomic region equaling an average of 1 SNP per 380 bp. No SNP were identified within the coding region of the gene, although many SNP or short indels are present surrounding the intron-exon boundaries. For *Tex11* 530 SNP were identified across ~187 kb equaling an average of 1 SNP per 352 bp. Three previously unreported nsSNP were identified within exons 1, 10 and 23. It has previously been estimated that 20–30% of nsSNP affect protein function [37,38]. The virtues of using these SNP as targets for the current study included the ease of assay development and potential functionality of SNP. Additionally, these SNP were located across a large genomic area (135.7 kb) spanning multiple exons of the candidate gene.

### AR SNP and indel validation by direct sequencing of animals with extreme SC

**Figure 1:**
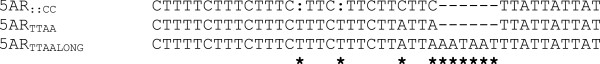
**Analysis of the 5′UTR region of AR.** Alignment of three haplotypes identified in the 5′UTR of the AR gene in the Brahman bull population. * highlights sites of variation across haplotypes.

Initial screening of selected SNP by PCR and sequencing within 5′UTR, intron-exon boundary regions and 3′UTR of the AR gene was performed using small panels of phenotypically extreme SC animals from within the Brahman bull population. The sequencing confirmed that 13 markers (12 SNP and 1 indel) previously identified bioinformatically were represented within the Brahman population. Furthermore, the sequencing exercise identified 3 markers (2 SNP and 1 indel) that were not observed in bioinformatic analyses (Table 1). Of particular interest were a cluster of 4 polymorphisms (2 SNP and 2 indel) within the 5′UTR region (X:88,621,605-88,621,617 bp). These markers formed three haplotypes subsequently designated as 5AR_::CC,_ 5AR _TTAA_ and 5AR_TTAALONG_ (Figure 1).

**Table 1:** Novel SNP or indels identified in the AR gene within the Brahman population using a small sample of high and low SC Brahman bulls

**SNP**	**Position on chr. X**^**a**^	**Gene Region**	**Base change B1-B2**	**Primer pair**^**#**^
1	88,621,617	5′UTR	C - A	For 5UTR + Rev exon1
2	88,621,614	5′UTR	C - A	“
3	88,621,609^*^	5′UTR	: - T	“
4	88,621,605	5′UTR	: - T	“
5	88,619,468	Intron 1	G - A	For exon 1 + Rev intron 1
6	88,491,830^*^	Intron 2	C - T	For Intron 1 + Rev Intron 2
7	88,449,481	Intron 2	T - G	For Intron 2 + Rev Intron 3
8	88,418,830	Intron 4	G - A	For Intron 4 + Rev intron 5
9	88,418,772	Intron 4	C - T	“
10	88,418,724	Intron 4	C - T	“
11	88,418,702	Intron 4	G - A	“
12	88,411,385	Intron 6	A - G	For Intron 5 + Rev intron 6
13	88,411823	Intron 6	A - G	For Intron 6 + Rev Intron 7
14	88,411595	Intron 7	C - A	“
15	88,411,276^*^	Intron 7	T - A	“
16	88,411,702	3 UTR	C - T	For Intron 7 + Rev 3 UTR

^a^Based up UMD3.1 Bovine Genome Assembly.

^*^Not identified in bioinformatic analyses.

^#^In naming of primers, “For” indicated a forward primer and “Rev” indicated a reverse primer.

### AR 5′UTR association study

Due to the difficulty in developing a high-throughput assay for this region, only a small number (n = 259) of Brahman bulls were characterised by direct sequencing with haplotype frequencies of 5AR_::CC,_ 5AR _TTAA_ and 5AR_TTAALONG_ being 59.1%, 36.3% and 4.6% respectively. Despite the relatively small sample size limiting the power of the subsequent association analysis, statistical analysis based upon the G > T SNP at position 88,621,617 across the 259 animals reveals that the SNP was associated with SC12, SC18 and SC24 with *P* values of 0.011, 0.0128 and 0.0144, respectively. This G > T SNP served as a tag SNP to the haplotypes due to perfect LD between these SNP.

A possible mechanism for the effect of the *AR* 5′UTR polymorphism may be changes to transcription factor binding sites across this region, as demonstrated through *in silico* studies (Table 2). Of particular interest is the presence of 4 putative SRY binding sites in 5AR_TTAA_ compared to only 2 comparable sites in each of 5AR_::CC and_ 5AR_TTAALONG_. SRY is a testis-expressed protein encoded by the sex-determining region on the Y chromosome that mediates male sex determination [39,40]. A previous study has shown that human SRY interacts with and negatively regulates *AR* transcriptional activity [41]. Our data suggests that those bulls with the 5AR_TTAA_ haplotype in the 5′UTR region of *AR* have a smaller SC at 12, 18 and 24 months. One hypothesis raised from this result is that the increased affinity for SRY in these animals leads to reduced transcription of the *AR* gene with downstream impacts upon rate of scrotal development and puberty.

**Table 2:** Types and frequencies of transcription factors putative binding sites in the Androgen Receptor

**AC (Reference)**	**Description**^**a**^	**Number of putative Sites**^**a**^		
		**5AR**_**::CC**_	**5AR**_**TTAA**_	**5AR**_**TTAALONG**_
M00028	HSF (Drosophila)	7	5	5
(Fernandes et al., 1994)				
M00101	CdxA	1	3	5
(Margalit *et al.*, 1993)				
M00029	HSF (Yeast)	4	5	7
(Fernandes et al., 1994)				
M00148	SRY	2	4	2
(Fernandes et al., 1994)				
M00137	Oct-1	1	3	5
(Verrijzer et al., 1992)				
M00094	BR-C Z4	1	2	1
(Vonkalm et al., 1994)				

^**a**^Types and frequencies of transcription factors putative binding sites in these regions were based upon *in silico* DNA transcription factor binding site prediction software TFSEARCH (http://www.cbrc.jp/research/db/TFSEARCH.html).

### Targeted AR and Tex11 SNP genotyping and association analysis by TaqMan® assay

Using TaqMan® assays, 980 Brahman and 619 Tropical Composite bulls were genotyped for 4 SNP including 3 SNP in *Tex11* (Tex11_ r38k, Tex11_g297d, Tex11_r696h) and a SNP in intron 4 of the *AR* gene (AR_In4) (Additional file 1: Table S1). As expected during allelic discrimination analyses, heterozygosity was not observed as these markers were present on the X chromosome of male samples. Statistical analyses demonstrated that three SNP (Tex11_ r38k, Tex11_r696h and AR_In4) were strongly associated with SC12, SC18 and SC24 in Brahmans with *P* values in the range 10^-7^-10^-14^ (Table 3). These associations were subsequently confirmed in the Tropical Composite bulls (Table 4). The SNP Tex11_g297d was also associated with PNS18 and PNS24 in Brahmans (Table 3), while all markers except for Tex11_g297d were associated with PNS18 and PNS24 in the Tropical Composite bulls (Table 4). Observed allele frequency for the A allele in Tropical Composite and Brahman bulls was 0.23 and 0.17 for Tex11_ r38k, 0.12 and 0.27 for Tex11_g297d, 0.23 and 0.17 for Tex11_r696h, and 0.16 and 0.52 for AR_In4, respectively.

**Table 3:** ***AR *** **and *Tex11 *** **SNP association analysis in the Brahman cattle population**

**Trait**	**SNP**	*** P***	**Allele**	**Effect**	**SE**	**%Va**
PNS18	AR1_In4	0.1159	T	−3.793	2.408	0.425
PNS18	Tex11_r38k	0.4506	G	2.387	3.153	0.097
PNS18	Tex11_g297d	0.0155	A	6.821	2.809	1.099
PNS18	Tex11_r696h	0.4933	G	2.168	3.168	0.077
PNS24	AR1_In4	0.7291	T	0.5082	1.467	0.014
PNS24	Tex11_r38k	0.0796	G	−3.456	1.968	0.374
PNS24	Tex11_g297d	0.0081	A	4.578	1.723	0.912
PNS24	Tex11_r696h	0.0906	G	−3.43	2.023	0.355
SC12	AR1_In4	0.0001	T	−0.5531	0.1405	3.249
SC12	Tex11_r38k	4.38 x10^-8^	G	1.038	0.1881	6.579
SC12	Tex11_g297d	0.4797	A	−0.119	0.1679	0.120
SC12	Tex11_r696h	1.30 x10^-7^	G	1.012	0.1902	6.037
SC18	AR1_In4	5.04 x10^-8^	T	−0.8811	0.1604	4.820
SC18	Tex11_r38k	8.25 x10^-14^	G	1.625	0.2142	9.427
SC18	Tex11_g297d	0.0862	A	−0.3299	0.192	0.540
SC18	Tex11_r696h	5.79 x10^-13^	G	1.582	0.2165	8.625
SC24	AR1_In4	4.64 x10^-10^	T	−0.9485	0.1506	5.506
SC24	Tex11_r38k	6.24 x10^-14^	G	1.544	0.2026	8.390
SC24	Tex11_g297d	0.0617	A	−0.3413	0.1825	0.570
SC24	Tex11_r696h	4.46 x10^-13^	G	1.507	0.2052	7.716

Significance (*P*), allele substitution effect (Effect) for the named allele of each SNP, its standard error (SE) and percentage of additive variance (%Va) explained by 3 SNP genotypes in *Tex11* and 1 SNP in *AR* on Scrotal Circumference (SC) at 12, 18 and 24 **months** of age and on the percentage of normal sperm (PNS) at 18 and 24 months of age.

**Table 4:** AR and Tex11 SNP association analysis in the tropical composite population

**Trait**	**SNP**	***P***	**Allele**	**Effect**	**SE**	**%Va**
PNS18	AR1_In4	0.0127	T	−6.384	2.551	5.646
PNS18	Tex11_r38k	0.0072	G	−6.181	2.29	6.911
PNS18	Tex11_g297d	0.5780	A	−1.655	2.992	0.299
PNS18	Tex11_r696h	0.0063	G	−6.266	2.282	7.169
PNS24	AR1_In4	0.0784	T	−4.137	2.347	2.368
PNS24	Tex11_r38k	0.0002	G	−7.838	2.101	11.102
PNS24	Tex11_g297d	0.0594	A	−5.127	2.715	2.863
PNS24	Tex11_r696h	0.0006	G	−7.31	2.121	9.746
SC12	AR1_In4	5.02 x 10^-6^	T	−1.427	0.3097	7.976
SC12	Tex11_r38k	4.00 x 10^-15^	G	−2.155	0.2669	23.754
SC12	Tex11_g297d	3.35 x 10^-5^	A	−1.517	0.3628	7.096
SC12	Tex11_r696h	2.22 x 10^-16^	G	−2.28	0.2691	26.837
SC18	AR1_In4	4.98 x 10^-7^	T	−1.565	0.3077	10.584
SC18	Tex11_r38k	4.88 x 10^-14^	G	−2.066	0.2674	24.086
SC18	Tex11_g297d	7.45 x 10^-4^	A	−1.222	0.3604	5.079
SC18	Tex11_r696h	1.44 x 10^-14^	G	−2.137	0.2705	26.009
SC24	AR1_In4	1.66 x 10^-7^	T	−1.548	0.2922	10.772
SC24	Tex11_r38k	3.03 x 10^-12^	G	−1.839	0.2579	19.853
SC24	Tex11_g297d	0.0066	A	−0.9469	0.3471	3.173
SC24	Tex11_r696h	2.18 x10^-12^	G	−1.865	0.2597	20.607

Significance (*P*), allele substitution effect (Effect) for the named allele of each SNP, its standard error (SE) and percentage of additive variance (%Va) explained by 3 SNP genotypes in *Tex11* and 1 SNP in *AR* on Scrotal Circumference (SC) at 12, 18 and 24 **months** of age and on the percentage of normal sperm (PNS) at 18 and 24 months of age.

### Genomic examination of frequency of taurine and indicine specific alleles

**Figure 2:**
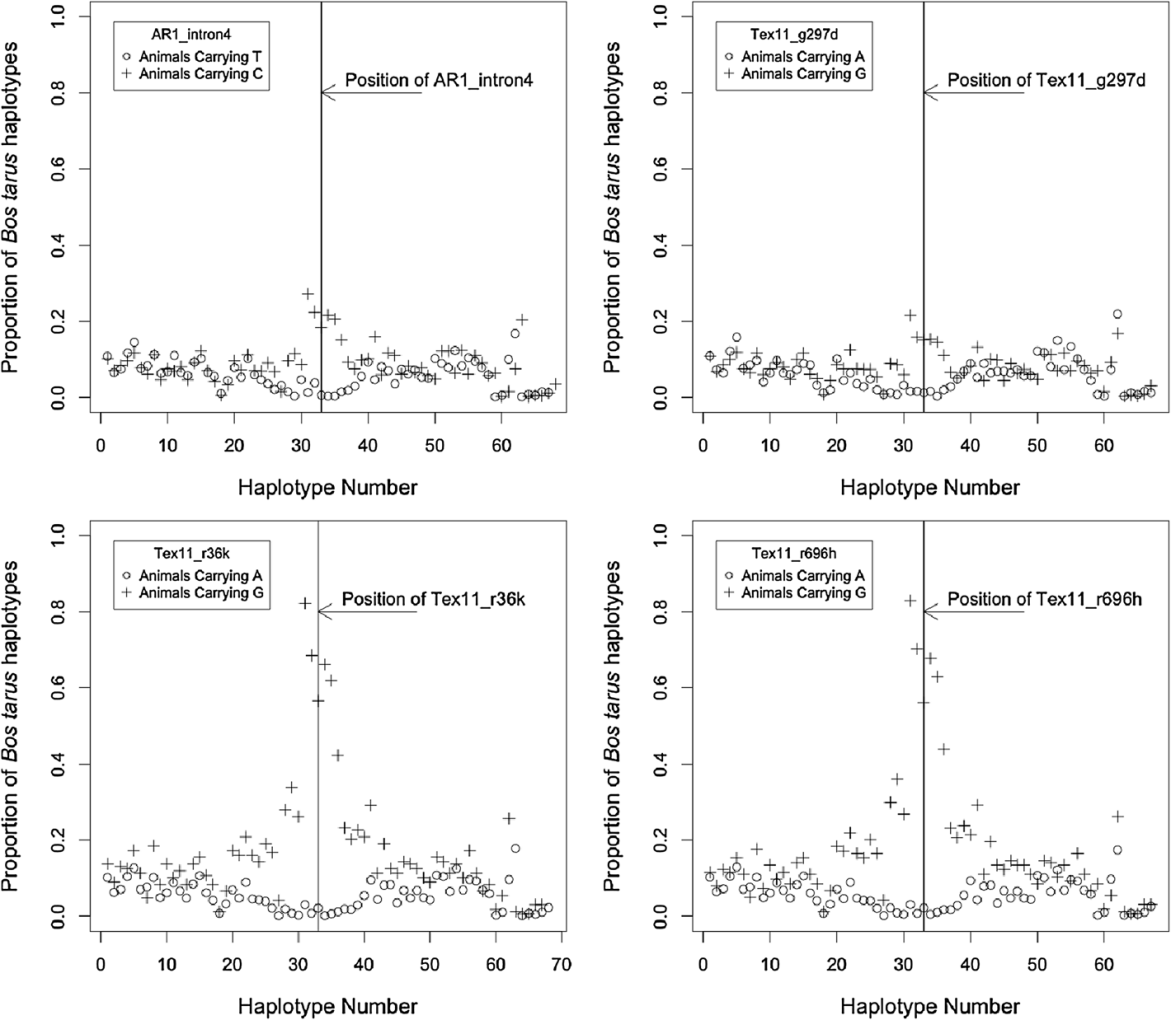
**Distribution of the proportion of *B. taurus *** **alleles across the X chromosome in the Brahman bull population based upon grouping based upon candidate marker alleles.**

Across the entire genome or entire chromosome X, cattle carrying the favourable Tex11_r38k allele for SC had 2.6% of *Bos taurus* component and bulls carrying the less favourable allele had 2.3%. When animals were grouped on the basis of allelic variants at the four SNP (AR_In4 [G > A], Tex11_g297d [A > G] Tex11_r38k [A > G], Tex11_r696h [A > G]), differences in the frequency of *Bos taurus* haplotypes were observed (Figure 2, Additional file 2: Table S2). For Tex11_r38k and Tex11_r696h, animals carrying the G allele had 67% of *Bos taurus* haplotypes (ranging from 82% to 57%) and animals carrying the A allele had 1.33% on average (ranging from 0.6% to 3%). For AR_In4, 18% and 3% of *Bos taurus* haplotypes were observed for those animals with G and A alleles respectively. For Tex11_g297d 16% and 3% of *Bos taurus* haplotypes were the average for bulls with G and A alleles respectively.

## Discussion

Several studies have been published on the role of polymorphisms in other candidate genes associated to pubertal traits in cattle. For example, polymorphisms in insulin like growth factor 1 (*IGF1*), gonadotropin-releasing hormone receptor (*GNRHR*) and testicular luteinizing hormone receptor (*LHR*) were associated with age at puberty in Angus male cattle, in Polish Holstein-Friesian cattle [42] and in Female Brahman cattle [43]. This current analysis is the first to implicate *Tex11* and *AR* as regulators of puberty onset in bovine bulls.

Previous studies have estimated that Australian Brahman cattle may contain up to 10% *Bos taurus* genes and that these chromosomal segments can have significant effects on body weight or other production traits [44]. Upon examination across the entire genome or entire chromosome X, cattle carrying the favourable Tex11_r38k allele for SC were found to have similar *Bos taurus* component when compared to bulls carrying the less favourable allele (2.6% and 2.3% respectively). However, when animals were grouped on the basis of allelic variants at the four SNP (AR_In4 [G > A], Tex11_g297d [A > G] Tex11_r38k [A > G], Tex11_r696h [A > G]), striking differences were found in the average frequency of *Bos taurus* haplotypes between these groups (Figure 2). The difference between groups was most pronounced around the Tex11_r38k and Tex11_r696h loci. For a 5 haplotype region (each haplotype containing 10 SNP), animals carrying the G allele, favourable for higher SC, had higher levels of *Bos taurus* haplotypes (67% on average) compared to those bulls carrying the A allele at these loci (only 1.33% on average). A similar but less pronounced effect was observed for AR_In4 (18% compared to 3%) and Tex11_g297d (16% compared to 3%). The increase in the proportion of *Bos taurus* haplotypes at approximately 70–100 Mb on chromosome X encompasses the candidate genes and the previously characterised QTL region associated with SC, PNS and other reproductive traits in both Brahman and Tropical Composite bulls. This suggests that this region of *Bos taurus* origin may have been retained in Brahmans and Tropical Composites through positive selection due to its favourable phenotypic outcomes including larger SC related to younger age at puberty and higher fertility.

## Conclusions

In this study, two putative candidate genes (*AR* and *Tex11*) were investigated for their association with SC and PNS measurements, recognised fertility traits in Brahman and Tropical Composite bulls. These genes were selected for this study because of their position, which mapped to reported QTL [29,30], and because of their known functions. Our results provide strong evidence for the biological role played by *AR* and in particular *Tex11* in male reproduction in cattle. These two genes emerge as strong candidate genes for explaining SC and PNS variation in tropically adapted cattle breeds such as Brahman and Tropical Composite, with varying degrees of taurine and indicine content. Evidence for recent positive selection of a favourable *Bos taurus* haplotype was shown and potential causative mutations were described, such as the nsSNP in *Tex11* and the 5’UTR SNP in *AR* that might affect SRY transcription factor binding sites. Knowledge about causative mutations merits confirmation and functional studies. These causative mutations can be used to improve animal breeding and selection as well as being potential models for male fertility issues in mammals.

## Methods

**Figure 3:**
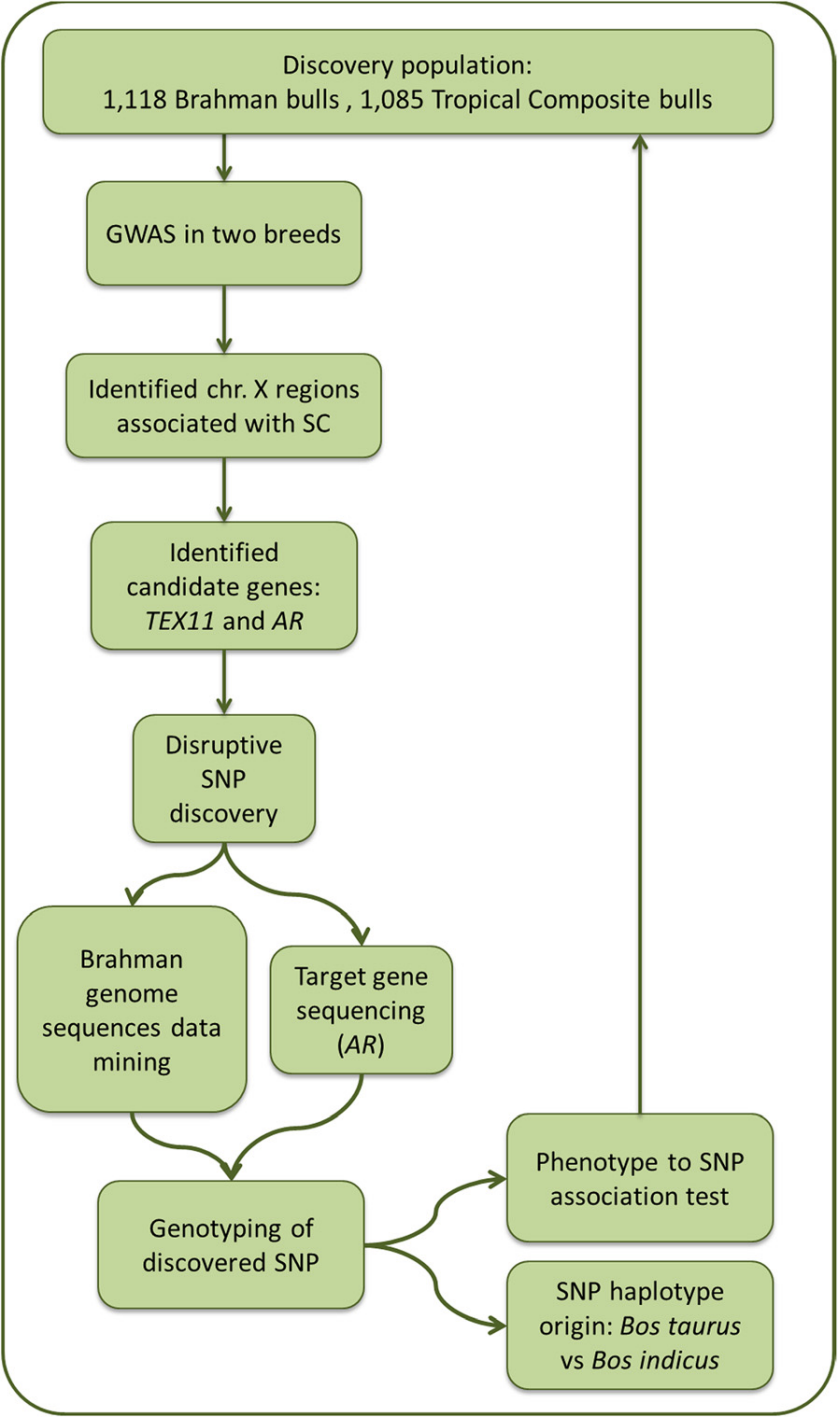
Flow of research procedures from genome-wide association discovery of associated genomic regions to test of novel single nucleotide polymorphisms located in candidate genes.

The flow of procedures that formed this study is summarized in the illustration provided (Figure 3) and detailed below.

### Animals

Animal Care and Use Committee approval was not required for this study because the data were obtained from existing phenotypic databases and DNA storage banks as described below.

Data from 1,118 Brahman bulls born between 2004 and 2008 were available for the current study. These bulls were the progeny of 55 sires from a larger Cooperative Research Centre for Beef Genetic Technologies (Beef CRC) breeding experiment to investigate genetics associated with whole herd profitability [12,45-48]. Data from 1,085 Tropical Composite bulls born between 2007 and 2009, developed with combinations of Belmont Red, Charbray, Santa Gertrudis and Senepol breeds that represent a genotype with 50% tropically adapted and 50% unadapted genetics were available to use in the current study [47]. These bulls were the progeny of 56 sires also from the Beef CRC. Experimental design, trait measurement details and quantitative genetic analyses of these Brahman and Tropical Composite populations have been described previously [12,47].

### Phenotypic data

Traits utilized in this study are components of male fertility: scrotal circumference (SC) at 12 (SC12), 18 (SC18) and 24 (SC24) months and percentage of normal sperm (PSN) at 18 (PNS18) and 24 (PNS24) months. These traits were measured at the bull breeding soundness evaluations (BBSE). Scrotal circumference was measured in centimetres using a standard metal tape [49]. As part of BBSE, bulls having a SC of 20 cm or more were subjected to electro-ejaculation. The percentage of normal sperm (PNS) was based on sperm morphology assessment which was carried out by a laboratory technician accredited by the Australian Cattle Veterinarians [29,30,49].

### Bioinformatic analyses of genome sequences

As part of the larger Beef CRC project, the genomes of sixteen Brahman bulls were sequenced at CSIRO Animal, Food and Health Sciences in St Lucia (unpublished data). These bulls were from the cohort of 55 sires used to generate the population used in GWAS analyses [26-28,30,42]. In brief, DNA from all bulls were size selected to the range 400–600 bp, DNA libraries for sequencing by reversible terminator chemistry were prepared for each animal separately using the standard Illumina kit, and 100 bp paired-end reads were collected using an Illumina HiScan sequencer. Raw data reads were analysed using the CASAVA 1.8 software package (http://www.illumina.com/software/genome_analyzer_software.ilmn), where reads were matched to the UMD3.1 Bovine Genome Assembly. A single BAM format file was created for each sire.

Using Samtools [36], raw genomic data covering the 60–94 Mb region of chromosome X were extracted for each of the 16 animals as individual bam files, and all were aligned to the *Bos taurus* UMD3.1 Bovine Genome Assembly. Variant calls were outputted in VCF format. Variant Effect Predictor (VEP) was used to predict the functional consequences of detected variants [50]. *In silico* prediction of DNA transcription factor binding sites was performed using TFSEARCH [51].

### Androgen receptor gene characterisation

The AR gene is highly conserved across populations, with no nsSNP detected by bioinformatic analyses. Classic PCR and sequencing methodologies across exon-intron boundaries were used to confirm novel non-coding SNP that may be experimentally valuable for further characterisation of this region in studied populations. For sequencing of the *AR* gene, DNA samples were selected from 17 bulls representing the extreme phenotypes for SC. Sequencing was performed for 10 animals with the highest SC measurements, high SC, and 7 animals with the lowest SC measurements, low SC. Primer design used the *Bos taurus* reference gene sequence (ENSBTAG00000022255). Primer pairs were designed to amplify a number of intronic regions immediately up- or downstream of known exons, with a full list of primer pairs in Additional file 3: Table S3.

PCR amplification was performed in an Eppendorf Master Cycler Gradient EP-S thermal cycler. PCR reactions (25 μl) contained 50–100 ng DNA, 1x GoTaq® PCR master mix (Promega, Australia) and 0.5 μM of each primer. The reaction profile used was 94 ^0^C for 2 mins, 35 cycles of 94°C for 10 s, primer-dependent annealing temperature (Additional file 3: Table S3) for 20s and 72°C for 2 mins, followed by a final extension at 72°C for 10 mins. PCR products were separated on 1% agarose gels with 1xTAE (Tris-acetate-EDTA) and visualized on UV transilluminator. PCR products with the expected size range (Additional file 3: Table S3) were sequenced with the forward and reverse primers using BIG DYE 3.1 terminator mix on an ABI 3130*xl* Genetic Analyser (Applied Biosystems, CA, USA). DNA sequence data were analyzed using Sequencher™ 4.1 (GeneCodes, MI, USA).

For a number of PCR products, deletions predicated the need for cloning of products prior to sequencing. When necessary, PCR products from individual animals were excised from the gel and purified using the QIAquick gel extraction kit (QIAGEN, Doncaster, VIC, Australia). PCR products were then cloned into pGem-Teasy vector (Promega, Australia). Putative positive clones were sequenced with M13 forward and reverse primers.

### Genotyping of selected SNP

Custom TaqMan® assays were developed for a novel non-coding SNP in the *AR* gene (AR_In4) located in the fourth intron, as well as 3 novel nsSNP spanning the *Tex11* gene (Tex11_ r38k, Tex11_g297d, Tex11_ r696h). Primers and probes were developed using the Custom TaqMan® Array Design Tool, and are listed in Additional file 1 Table S1.

Genotyping was performed by allelic discrimination using custom TaqMan® SNP genotyping assays, following the manufacturer's instructions. Briefly, 5 μl PCR reactions were carried out containing 2.5 μl of TaqMan® Universal PCR Master Mix (Applied Biosystems, New Jersey, USA), 10 ng of DNA template and 0.25 μl of TaqMan® assay primers and FAM/VIC labelled probes by Applied Biosystems as Assays-by-Design™ (Applied Biosystems, Foster City, CA, USA). All thermal cycling experiments were performed in 384 well plates on a Gene Amp 9700 (Applied Biosystems). Amplification conditions consisted of 50°C for 2 min, 95°C for 10 min followed by 40 cycles of 95°C for 30 s and 60°C for 1 min, and finally 25°C until removed from the thermal cycler. End-point reads were then performed on the Applied Biosystems ViiA™ 7 Real-Time PCR System, and allelic discrimination analysis was performed using ViiA™ 7RUO software (Life Technologies, CA, USA).

### Statistical analyses

The association of each SNP with SC12, SC18, SC24, PNS18 and PNS24 was examined for genotyped Brahman bulls using a mixed model analysis of variance with ASREML software [52]. The analysis performed was similar to those performed in previous GWAS and quantitative genetic analyses [12,29,30]. Briefly, the mixed model can be written as follows:

$$y_{i}=\mathrm{X\beta}+\mathrm{Z\mu}+S_{j}a_{j}+e_{i}$$

Where *y*_*i*_ represents the phenotypic measurement for the *i*th animal, *X* is the incidence matrix relating fixed effects in β with observations in y, *Z* is the incidence matrix relating to random additive polygenic effects of animal in *μ* with observations in y and *Sj* is the observed animal genotype for the *j*^th^ SNP (coded as 0, 1 or 2 to represent the number of copies of the B allele), *α*_*j*_ is the estimated SNP effect, lastly *e*_*i*_ is the random residual effect. The same fixed effects were used for each trait. These fixed effects included contemporary group (animals born in the same year and raised together) and a second term that was the interaction of year and month of birth.

The percentage of the genetic variance accounted by the *j*^-th^ SNP was estimated according to the formula $\%V_{j}\;=\;100\cdot \frac{2p_{j}q_{j}a_{j}^{2}}{\sigma_{g}^{2}}$ where pi and qi are the allele frequencies for the *j*^-th^ SNP estimated across the entire population, *a*_*j*_ is the estimated additive effect of the *j*^-th^ SNP on the trait under analysis, and *σ*_*g*_^2^ is the REML estimate of the (poly-) genetic variance for the trait.

The origin of haplotypes on the X chromosome was determined by using the method described by Bolormaa et al. [44]. This method classifies haplotypes as either *Bos taurus* or *Bos indicus* in origin depending on their relative frequency in a reference population. In brief, haplotypes of 10 consecutive SNP were built and their frequencies calculated in 1,105 Brahman bulls (reference population for *Bos indicus*) and 3,666 Angus, Hereford, Murray Grey and Shorthorn bulls (reference population for *Bos taurus*) of the Beef CRC. The probability that each segment was of *Bos taurus* origin (*b*_*i*_) was calculated using the following formula:

$$\;b_{i}=\frac{p_{\mathrm{Bti}}}{p_{\mathrm{Bti}}+p_{\mathrm{Bii}}}$$

Where *p*_*Bti*_ is the frequency of haplotype segment *i* in *Bos taurus* animals and *p*_*Bii*_ is the frequency of haplotype segment *i* in *Bos indicus* (Brahman) animals. Segments were classified as *Bos taurus* if *b*_*i*_ > 0.6 and *Bos indicus* if *b*_*i*_ < 0.4. Haplotype segments with *b*_*i*_ between 0.4 and 0.6 were classified as undetermined origin.

### Genomic examination of frequency of taurine and indicine specific alleles

To determine whether there was a general increase in the proportion of *Bos taurus* alleles across the genome or X chromosome in animals carrying the G allele of the Tex11_r38k SNP, the percentage of *Bos taurus* in each animal was estimated using either all 50 k autosomal SNP or all X chromosome SNP from the 50 k chip, using Admixture [53]. The data set used to estimate taurine content was 3,666 *Bos taurus* animals (Angus, Shorthorn Murray Grey and Hereford) and 1,032 Brahman bulls, all extracted from The Beef CRC database, with 50 k genotyping detailed before [29,30,54]. The mean and standard deviation of *Bos taurus* proportion was then calculated and used to compare animals with the A or G alleles of Tex11_r38k.

### Availability of supporting data

New SNP were submitted to NCBI and the list of SS reference numbers is provided in the additional material section (Additional file 4). Sequence data was submitted to the 1000 bull genomes project, which is a collection of sequence data, intended as a resource for the bovine research community (http://www.1000bullgenomes.com/).

## Competing interests

The authors declare that they have no competing interests.

## Authors’ contributions

REL, SAL, NTL, SSM, LD and MRSF participated in study design. NTL, LD, REL performed experiments and analysed data. SSMcW provided support in the bioinformatic analysis. MK and YL performed statistical analyses. WB, RJB and BEH contributed genomic data for bioinformatic analyses. REL drafted the manuscript. All authors aided in the manuscript preparation.

## Supplementary Material

Additional file 1: Table S1 SNP genotyped and nucleotide sequences of primers and probes used in TaqMan® Assays. Click here for file

Additional file 2: Table S2 Mean and Standard deviation of estimated *B. taurus* content of animals carrying the A and G allele of Tex11_r36k calculated using different marker sets from full autosomes (40 k) through subset of X chromosome (628). Click here for file

Additional file 3: Table S3 Forward and reverse primers used to amplify the intron-exon boundaries, 5′UTR and 3′UTR regions of the AR gene. Click here for file

Additional file 4 Supplementary list of SS reference numbers for new polymorphisms as provided by NCBI. Click here for file
