# Supplementary material for: Evidence for positive selection of taurine genes within a QTL region on chromosome X associated with testicular size in Australian Brahman cattle
Source: BMC Genet. 2014 Jan 10;15:6. doi: 10.1186/1471-2156-15-6 (PMC3893399; doi:10.1186/1471-2156-15-6)
Supplement: Additional file 1: Table S1 — SNP genotyped and nucleotide sequences of primers and probes used in TaqMan® Assays. [file 1471-2156-15-6-S1.docx]

**Table S1:** SNPs genotyped and nucleotide sequences of primers and probes used in TaqMan® Assays.

| Gene ID | Genomic Position^a^ | SNP bases | Primer (5' – 3') | Probe (5' – 3') |
| --- | --- | --- | --- | --- |
| AR_In4 | X:88,418,702 | G>A | AGTTAATTCATTGTAAGCAGTCAGTGTTAGAT  AAAGAGATGGTAACGGAGGCAAAA | VIC-CCATTGAAAGAGGATAATA  FAM-CCATTGAAAGAGAATAATA |
| Tex11_ r38k | X:85,042,933 | G>A | CGGCAATTGATAGACTCTTCATGGA  CTGAGAATCCTGTATTTCAGCCATA | VIC-AGGAAATATCAACAGAGAGTC  FAM-TAGGAAATATCAACAAAGAGTC |
| Tex11_ g297d | X:85,115,764 | G>A | ATCCAGTTGGGCTTTTCTTAAAGGT  TGTACTTTTCACACACAGAGATGTTACTT | VIC-AGGGTGAAATAGGTAATGAA  FAM-AGGGTGAAATAGATAATGAA |
| Tex11_ r696h | X:85,178,633 | G>A | TGCTTCCTAAATCGTGCACTTGA  TCCCACTGTACCTGTTGTTTTCAG | VIC-TCTGATCCGTAAATGC  FAM-TCTGATCCATAAATGC |

^a^ based upon *B .taurus* UMD3.1 Bovine Genome Assembly
