# Supplementary material for: Evidence for positive selection of taurine genes within a QTL region on chromosome X associated with testicular size in Australian Brahman cattle
Source: BMC Genet. 2014 Jan 10;15:6. doi: 10.1186/1471-2156-15-6 (PMC3893399; doi:10.1186/1471-2156-15-6)
Supplement: Additional file 2: Table S2 — Mean and Standard deviation of estimated B. taurus content of animals carrying the A and G allele of Tex11_r36k calculated using different marker sets from full autosomes (40 k) through subset of X chromosome (628). [file 1471-2156-15-6-S2.docx]

**Table S2:** Mean and Standard deviation of estimated *B. taurus* content of animals carrying the A and G allele of Tex11_r36k calculated using different marker sets from full autosomes (40k) through subset of X chromosome (628).

| Markers | A | | | G | | |
| --- | --- | --- | --- | --- | --- | --- |
| Autosomes | 0.0258 | + | 0.0249 | 0.0262 | + | 0.0228 |
| Full X | 0.0023 | + | 0.0150 | 0.0023 | + | 0.0086 |
