# Supplementary material for: Evidence for positive selection of taurine genes within a QTL region on chromosome X associated with testicular size in Australian Brahman cattle
Source: BMC Genet. 2014 Jan 10;15:6. doi: 10.1186/1471-2156-15-6 (PMC3893399; doi:10.1186/1471-2156-15-6)
Supplement: Additional file 3: Table S3 — Forward and reverse primers used to amplify the intron-exon boundaries, 5′UTR and 3′UTR regions of the AR gene. [file 1471-2156-15-6-S3.docx]

**Table S3:** Forward and reverse primers used to amplify the intron-exon boundaries, 5’UTR and 3’UTR regions of the AR gene.

| Transcribed Region | Primer name | Primer sequence | Tm | Product size (bp) | |
| --- | --- | --- | --- | --- | --- |
| 5’ UTR | For 5’ UTR  Rev_exon 1 | CGG AGC TGC CTT TTC TTC TT  GTA GAC CCT CCC CAG CCC TA | 60 | 1002 bp | |
| Exon 1 | AR_For_UTR  AR_Rev-Exon | TCA GCA AGA AAA GGG GAG CA  GAG GAG AGA CTT ACC GCA TGT | 60 | 1098 bp | |
| exon 1B | For_exon1  Rev_exon1 | AAC TTC TTC AGC AGC AGC AAC  GAG TGT GCC AGG AAG AGG AG | 60 | 744 bp | |
| exon 2 | For_intron 1  Rev_intron2 | ATC GCC ATA GGT CTC CCT TT  CAC TTC CTT TCT GGG GTT GA | 60 | 1007 bp | |
| exon 3 | For_intron2  Rev_Intron3 | TCA TGC ATC AAA CGT GGA CT  CTC TGC TTG GCA GCC TAG TT | 60 | 1093 bp | |
| exon 4 | For_intron3  Rev_intron4 | ATT TAG GCA GAG CCC AAT CC  CCA ACC CCA TGT TCC TTC TA | 60 | 1011 bp | |
| exon 5 | For_intron4  Rev_intron5 | GCA TGG GCT TTG GAA TAA GA  GTT GCC CAG AGT GAA GAA GG | 60 | 912 bp | |
| exon 6 | For_intron5  Rev_Intron6 | TGG GAA TCC TGG TGT CCT TA  GCT GCC AAA TAA GCC TTT TC | 58 | 970 bp | |
| exon 7 | For_intron6  Rev_Intron7 | CCT ATG GGG GAG AAA GGA GA  CTT TTC CCC AGC ACA CAC AC | 59 | 981 bp | |
| exon 8 | For_Intron7  Rev_3UTR | CTG GGG AAA AGG AAC ACT CA  GGG CTT GCA ATT TTC ACC T | 57 | 970 bp |  |
